# The Associations between Objectively Measured Physical Activity and Physical Function in Community-Dwelling Older Japanese Men and Women

**DOI:** 10.3390/ijerph19010369

**Published:** 2021-12-30

**Authors:** Harukaze Yatsugi, Tao Chen, Si Chen, Xin Liu, Hiro Kishimoto

**Affiliations:** 1 Department of Behavior and Health Sciences, Graduate School of Human-Environment Studies, Kyushu University, 744 Motooka Nishi-ku, Fukuoka 819-0395, Japan; haru19920424@gmail.com (H.Y.); liuxinjp1992@gmail.com (X.L.); 2 Sports and Health Research Center, Department of Physical Education, Tongji University, Shanghai 200092, China; chentwhy@tongji.edu.cn; 3 School of Nursing and Rehabilitation, Cheeloo College of Medicine, Shandong University, 44 West Wenhua Road, Jinan 250012, China; chensi@sdu.edu.cn; 4 Faculty of Arts and Science, IC15, Kyushu University, 744 Motooka Nishi-ku, Fukuoka 819-0395, Japan

**Keywords:** physical activity, physical function, tri-axial accelerometer, older adult

## Abstract

Objective: The relationships between physical activity (PA) and physical function (PF) among older Japanese adults have been examined before, with some studies reporting that moderate-to-vigorous physical activity (MVPA) is positively associated with PF. However, it is unclear whether the observed associations differ between men and women. In this study, we investigated the associations of objectively measured MVPA, light physical activity (LPA), and sedentary time (ST) with different PF levels in older Japanese men and women. Subjects and Methods: A total of 810 community-dwelling subjects aged 65–75 years were investigated (52.1% women, 47.9% men). The PF variables included grip strength, one-leg standing, usual and maximum walking speeds, and chair-standing time. PA (MVPA, LPA, and ST) and the number of steps taken daily were assessed for seven consecutive days by a tri-axial accelerometer. We determined the sex-specific quartiles of MVPA, LPA, and ST and analyzed their associations with physical function in separate models for each exposure measure. Results: In the crude analysis, MVPA was significantly associated with all PF variables in the men, and with the usual walking speed, max. walking speed, and chair-standing time in the women. Neither LPA nor ST was significantly associated with any PF variables. After adjusting potential confounding factors, significant associations between MVPA and usual walking speed remained in the men and women. Conclusions: Only greater moderate-to-vigorous physical activity (MVPA) was significantly associated with higher levels of PF variables in both men and women. Thus, time spent in moderate-to-vigorous physical activity (MVPA) can help older adults maintain or improve their physical function.

## 1. Introduction

Physical activity (PA) is increasingly recognized as a factor that can decrease the risks of developing various diseases [1,2,3,4,5]. In older adults, their physical function (PF) is a crucial component of health, and a decline in PF is closely related to disability and mortality [6,7,8]. The Japanese Physical Activity Guideline for Health 2013 was implemented to encourage healthy activities among individuals of all ages, and Japan’s PA guidelines were subsequently designed to help the Japanese population increase their PA in daily life to improve and maintain their health [9]. However, these guidelines were standardized based on the findings of a meta-analysis that included four studies of western populations with self-reported PA [10,11,12,13]. It is thus necessary to objectively measure PA and discuss Japan’s PA guidelines’ normative values in relation to the objective measurements [14]. Several studies have described PA and PF values in older Japanese adults, but their results differed between men and women; in addition, the relationships observed differed from those described in other countries [15,16,17,18,19,20].

PF components were included in those studies, but different levels of PF may indicate different things. For example, a study conducted in Japan mentioned handgrip strength, usual gait speed, maximum gait speed, the timed up-and-go test, and one-leg standing with eyes open; another study mentioned five sit-to-stand measurements, one-leg standing with eyes open, the timed up-and-go test, usual walking speed, and the choice-stepping reaction time [8,21]. Because of the differences in these studies’ PF components, no unified conclusion can be drawn. One of these studies [8] concluded that maintaining daily life PA (steps) outside an exercise program is essential for improving walking ability, and the other [21] concluded that replacing even small amounts of sedentary behavior (SB; e.g., watching TV and working at a desk) with moderate-to-vigorous physical activity (MVPA; e.g., brisk walking and exercise/sport) may contribute to improvements in physical function in older adults.

At this juncture, objectively measured values related to PA and PF among older Japanese are still insufficient. Although relationships between PA and PF in older Japanese adults have been described, certain points remain unclear (e.g., only steps measured by a pedometer used as a PA variable, not evaluating men and women separately, etc.) [8,21]. We conducted the present study to examine the associations between objectively measured moderate-to-vigorous physical activity (MVPA), light physical activity (LPA), and sedentary time (ST) with PF by sex in older Japanese community-dwelling adults.

## 2. Subjects and Methods

### 2.1. Study Subjects

This study was based on the Itoshima Frail Study (2017) conducted in the city of Itoshima in Japan’s Fukuoka Prefecture. Of approximately 10,000 older residents of Itoshima aged 65–75 years who were not certified as requiring nursing care by the national long-term care insurance system, 5000 were randomly selected for this study according to their residential area, sex, and age. Questionnaires were mailed to these individuals along with an invitation for further assessments. Of the 5000 individuals we contacted, 1631 submitted questionnaires, and 949 participated in further assessments [19]. A final total of 810 participants (422 women (52.1%), 388 men (47.9%)) with valid data were included in the final study sample. This study was approved by the Institutional Review Board of Kyushu University, Japan. All subjects provided written informed consent to participate.

### 2.2. Physical Activity and Sedentary Time Measures

A tri-axial accelerometer objectively measured the subjects’ PA and ST (Active Style Pro HJA-350IT, Omron Healthcare, Kyoto, Japan). The subjects were instructed to wear the accelerometer on the right or left side of their waist for seven consecutive days and remove it only before going to bed or participating in water activities [20]. Data were recorded in 1-min epochs. The accuracy of the intensity estimated by the Active Style Pro has been validated with the Douglas bag method [22]. The use of a tri-axial accelerometer to assess PA and ST also allowed for a more accurate estimate of activity intensity compared with that of a conventional uni-axial accelerometer [22]. The technical specifications and data acquisition system for the Active style Pro have been reported [22,23].

We defined the non-wear time as ≥60 consecutive min of no activity, i.e., estimated activity intensity <1.0 metabolic equivalents (METs) with an allowance for 2 min of activities with an intensity up to 1.0 METs [15,20,24,25]. The data of only the subjects with ≥4 valid wear days (≥10 h of wear time/day) were included in the analysis [26]. The cut-off values used to define the amounts of time spent in ST, light-intensity physical activity (LPA), and MVPA were as follows: ≤1.5 METs for ST, 1.6–2.9 METs for LPA, and ≥3.0 METs for MVPA [20].

### 2.3. Physical Function Measurements

Five PF variables were objectively measured: maximum handgrip strength, one-leg-standing time, usual walking speed, maximum walking speed, and five-repetition sit-to-stand (chair-standing time) [27]. Briefly, the subjects’ handgrip strength was measured using a digital grip strength meter (Grip D, TKK-5401, Takei Scientific Instruments, Niigata, Japan). The measurements were performed twice on each hand alternately, with the maximum value adopted as the representative grip strength value [28]. The one-leg-standing time was measured with the subject standing barefoot with both eyes open and raising either the left or the right foot. The length of time before the subject touched the floor or wall was measured (for up to 120 s). Left and right measurements were taken once, with the longest time being adopted as the representative value for one-leg-standing time [29]. The 5-m gait speed was measured by having the subject walk straight from the starting point at his or her usual walking speed (UWS) and maximum walking speed (MWS). The duration from one foot crossing the 3-m point (the beginning of the measurement section) to crossing the 8-m point (the end of the measurement section) was determined. The measurements were performed twice, with the fastest time used as the representative value for the 5-m gait speed [29]. The chair-standing (motion) time was measured with the subject’s feet placed at approximately shoulder width, going from a seated position to standing in front of the chair. The time required to repeat this motion five times was measured [30].

### 2.4. Other Measures

Each subject’s body weight (kg) and body height (m) were measured using standard protocols with the subject in light clothing and without shoes. The subjects’ body mass index (BMI) values were calculated as weight in kilograms divided by height in meters squared. Socio-demographic characteristics including age, sex, years of education, living alone (yes/no), smoking (current smoker or not), and drinking (current drinker or not) were collected via a questionnaire. Polypharmacy was defined as taking five or more prescription medications (yes/no) [19].

Frailty status was assessed by a Japanese Fried Frailty Phenotype (FFP) scale, which includes shrinking, weakness, exhaustion, slowness, and low physical activity. The total score ranges from 0 to 5 points, and in the present study a score of 0 was considered to indicate robust participants, 1 and 2 as pre-frailty, and 3–5 as frailty. We used the same definition of Frailty status (cut-off points) referred to in a previous Japanese study [31].

### 2.5. Statistical Analyses

The descriptive data are presented as the mean (standard deviation (SD)) or median (Inter quartile range (IQR)). Student’s *t*-test was used to examine the significance of differences in PA and PF between the men and women. The χ^2^ (Chi-squared) test was used to examine the significance of differences in frequency (numbers of subjects). The associations between MVPA, LPA, ST, and the subjects’ PF were examined in an earlier investigation by linear regression, and the sex-specific quartiles of MVPA, LPA, and ST were defined and their associations with PF were examined in separate models for each exposure measure [32]. In the present study, we used analysis of variance (ANOVA) in Table 1, Table 2 and Table 3 and an analysis of covariance (ANCOVA) in Table 4 to examine the relationships between quartile groups related to PA and PF. Tests for trend across quartiles of each exposure were examined by assigning ordinal numbers (0, 1, 2, 3) to each exposure quartiles into the models. Additionally, the logarithmic conversion was used for MVPA, one-leg standing, and chair-standing time to examine the significance of differences because their distributions were skewed. We also observed similar results when raw data were used, thus non-transformed data were presented for easy interpretation. Model 1 adjusted for age, BMI, self-rated health, smoking habit, alcohol intake, osteoporosis, hypertension, hyperlipidemia, diabetes, stroke, heart disease, years of education, and accelerometer-wearing time. Model 2 adjusted for Model 1 and significant PF variables among the men and women, respectively. Because usual walking speed (UWS) and maximum walking speed (MWS) are strongly correlated, they were not adjusted by each walking speed. In addition, if other PF variables showed significance for both UWS and MWS, they were adjusted only by UWS. P for trend was tested to examine probability of each mean of variable (Table 2 and Table 3) and least square mean of variable (Table 4). All statistical analyses were conducted using SAS ver. 9.4 (SAS, Cary, NC, USA). The significance level was set at two-sided α = 0.05.

## 3. Results

### 3.1. The Characteristics of the 810 Older Community-Dwelling Japanese Subjects

Table 1 summarizes the characteristics of the subjects. The mean age of all 810 subjects was 70.9 years. Women accounted for 52.1% (*n* = 422) of the subjects, and men comprised 47.9% (*n* = 388). The men’s mean BMI (23.3 kg/m^2^) was significantly higher than the women’s (22.5 kg/m^2^). The men’s values of alcohol intake and smoking habits were significantly higher than the women’s. The women’s rates of osteoporosis and hyperlipidemia were significantly higher than the men’s, but the percentage of men with diabetes was significantly higher than that of the women. The number of years of education was significantly longer among the men than the women.

Regarding the PA variables, the men exhibited significantly high ST (*p* < 0.001) and significantly low LPA (*p* < 0.001) compared to the women. In contrast, the women exhibited significantly higher accelerometer wearing time (*p* < 0.0001), MVPA (*p* < 0.048), and LPA (*p* < 0.0001) compared to the men.

Regarding the PF variables, the men showed significantly higher grip strength and maximum walking speed (*p* < 0.0001), whereas the women exhibited significantly higher usual walking speed (*p* = 0.04).

### 3.2. The Associations between Physical Activity and Physical Function

Table 2 shows the associations between quartiles of PA variables and PF variables only in the men. Only MVPA was significantly associated with all PF variables in the men: grip strength (GS), one-leg standing (OS), usual walking speed (UWS), maximum walking speed (MWS), and the chair-standing time (CT). Table 3 shows the associations between the quartiles of PA variables and PF variables only in the women, and here, too, only MVPA was significantly associated with PF variables: usual walking speed, maximum walking speed, and chair-standing time.

Table 4 summarizes the differences in PF by quartiles of MVPA. After adjusting for Model 1 and Model 2, significant associations between MVPA and usual walking speed remained for both the men and the women. After adjusting for Model 2, significant associations between MVPA and grip strength were observed only among the women; likewise, after adjusting for Model 1 and Model 2, significant associations between MVPA and maximum walking speed were observed only among the women. Conversely, after adjusting for Model 1, significant associations between MVPA and chair-standing time were observed only among the men.

## 4. Discussion

We sought to determine the relationship between PA and PF in older Japanese community-dwellers, and the results of our analyses of the 810 subjects revealed that only the subjects’ MVPA was significantly associated with PF variables (in both the men and the women), and that the subjects’ LPA and ST data were not significantly associated with PF variables.

### 4.1. The Associations between Physical Activity and Physical Function 

MVPA was significantly associated with PF variables in the men and women, but LPA and ST were not. All of the PF variables (max. handgrip strength, one-leg-standing time, usual walking speed, max. walking speed, and chair-standing time) were significantly associated with MVPA in the men. In the women, walking speed (both usual and maximum), and chair-standing time were significantly associated with MVPA. Although LPA accounted for a substantial fraction of the total PA time (LPA + MVPA), only MVPA was significantly associated with PF variables. Significant differences in MVPA, LPA, and ST between the men and women were observed. Our results also suggested that these significant differences might have been affected by significant differences in the values of PF variables. A high-impact exercise program was reported to improve older males’ one-leg standing time, muscle function, and muscle thickness [33]. Our present findings suggest that MVPA in particular may affect short-time muscle exertion in older men.

### 4.2. The Associations between MVPA and Physical Function 

The significant relationship observed herein between MVPA and PF is in agreement with the findings of another study of Japanese subjects [8]. Other investigations in countries other than Japan have only described a significant association between MVPA and PF [34,35]. A strong association between higher physical capability and higher MVPA has been reported, but no evidence of independent associations with objectively measured sedentary time has been found [32].

In the present study, the women’s time spent in MVPA was significantly higher than that of the men (Table 1). However, we cannot draw any conclusion regarding the direction of a cause-and-effect relationship between MVPA and usual walking speed, due to the study’s cross-sectional design. The MVPA time might be affected by the subjects’ usual walking speed, which would be reflected in their activities in daily life. Some PF variables were significantly different between the men and the women, but the men and women had one result in common, i.e., their usual walking speed was significantly associated with their MVPA. Our findings suggest that the subjects’ time spent in MVPA in their daily lives was closely related to their usual walking speed, and that there were significant differences between the men and the women in this regard. A longitudinal study of rural community-dwelling older adults concluded that ‘usual gait speed’ was associated with frailty status, institutionalization, and mortality, and the study’s authors noted the necessity of considering a gait-speed cutoff value based on sex-specific quartiles to prevent misclassification in sarcopenia and frailty diagnoses [36]. We suggest that the MVPA time might be as effective as the usual walking speed for investigations of methods that can improve or maintain daily activity in older adults.

Now, the Japanese Physical Activity Guideline recommends that adults aged 18–64 years should engage in MVPA for 4 METs·hours/week (h/wk) and that older adults (≥65 years) should engage in PA regardless of its intensity for 10 METs·h/wk. At this juncture, Japan’s PA guidelines do not mention a recommended intensity of PA and ST for older adults [9]. We investigated the number of older Japanese engaged in MVPA at 10 METs·h/wk, and we observed that all of the subjects examined met this guideline and >70% of them met it by engaging in MVPA [20]. In light of the previous and present findings, we suggest that older Japanese should engage in PA in consideration of its intensity. Our present results indicate that among older adults, engaging in MVPA could be effective for maintaining or improving their PF.

### 4.3. The Associations between Physical Function and LPA and ST 

LPA and ST were not significantly associated with PF in our subjects, and this result agrees with that of an earlier investigation [8]. A recent systematic review and meta-analysis demonstrated the relative impact of a higher duration, intensity, and frequency of PA and less ST on muscle strength and muscle power, thus providing a foundation for informing interventions; it was also noted that absolute quantification is a priority for future lifestyle guidelines and the management of modifiable risk factors [37]. However, a cross-sectional study of older Japanese adults mentioned the possibility that spending less time in LPA might result in spending more time in ST [15]. In light of these findings, we speculate that achieving a higher percentage engaged in LPA or a lower percentage of ST are not sufficient for improving PF, and we suggest that engaging in a higher percentage of MVPA is a reasonable strategy for improving PF.

### 4.4. Implications of the Present Findings

Our analyses revealed that only MVPA was the most important factor for maintaining PF among older adults. It has been indicated that using various objective measures such as higher PA and lower SB were associated with greater muscle strength and muscle power among older adults, and MVPA was the most frequently reported measure that was often positively associated with muscle strength and power; this was an anticipated finding in the present study as MVPA is a strong determinant and predictor of health outcomes [37]. As noted above, the recommended intensities of PA and ST are not mentioned in Japan’s PA guidelines for older adults [9]. LPA may be associated with self-reported health and/or the mental health of older adults, but LPA may not be intense enough to be related to their physical function [8]. Our present results indicate that focusing on MVPA might be valid for maintaining or improving the physical functioning of older adults (both men and women), and we suggest that the Japanese PA guidelines for older adults should mention the intensity of PA in a future revision.

### 4.5. Study Strengths and Limitations

The strengths of this study include the relatively large Japanese population (>800 people) aged 65–75 years and the use of a tri-axial accelerometer to assess PA and ST, which provides a more accurate estimate of activity intensity than questionnaires. Our findings provide details of objectively measured physical activity, sedentary time, and physical function for older men and women in Japan.

Some limitations should be considered when interpreting our findings. (1) The recognized limitations of accelerometers include their inability to detect some types of PA (e.g., water activities and cycling) and distinguish between postures. The Active Style Pro HJA-350IT might underestimate MVPA among older adults [38]. (2) Almost all of our subjects were without long-term care and assistance; they might therefore have been relatively more active and motivated than the general population [20]. (3) The cross-sectional design of this study precludes the ability to examine the predictive ability to make causal inferences [19]. (4) The PF variables did not include endurance (aerobic) capacity or upper limb strength, although data of aerobic capacity or upper limb strength are more accurate for judging physical function and physical fitness.

## 5. Conclusions

Only moderate-to-vigorous physical activity (MVPA) was significantly associated with PF variables in both the older Japanese men and women in this study. Engaging in more moderate-to-vigorous physical activity can help older adults maintain or improve their physical function.

## Figures and Tables

**Table 1:** The subjects’ characteristics.

Covariable	Men (*n* = 388)	Women (*n* = 422)	*p*-Value
Age, years	70.9 (3.2)	70.9 (3.1)	0.997
BMI, kg/m^2^	23.3 (2.9)	22.5 (3.3)	**<0.0001**
Self-rated health, good, numbers	355 (91.5)	377 (89.3)	0.30
Alcohol intake, every day, numbers	211 (54.4)	36 (8.5)	**<0.0001**
Smoking habit, every day, numbers	44 (11.3)	5 (1.2)	**<0.0001**
Pre-frailty or Frailty, numbers	162 (41.8)	173 (41.0)	0.83
Osteoporosis, numbers	4 (1.0)	63 (14.9)	**<0.0001**
Hypertension, numbers	154 (39.7)	175 (41.5)	0.61
Hyperlipidemia, numbers	90 (23.2)	175 (41.5)	**<0.0001**
Diabetes, numbers	72 (18.6)	45 (10.7)	**0.001**
Stroke, numbers	20 (5.2)	13 (3.1)	0.14
Heart disease, numbers	49 (12.6)	36 (8.5)	0.057
Education history, years	13.6 (2.5)	12.3 (2.1)	**<0.0001**
Accelerometer wearing time, min/day	817.2 (86.5)	863.6 (89.4)	**<0.0001**
	816.4 (752.3–875.7)	868.5 (805.3–919.5)	
Steps	6043 (2989)	5421 (2554)	**0.0015**
	5692 (3921–7757)	4923 (3549–6891)	
MVPA, min/day	50.4 (31.9)	55.4 (33.2)	**0.048**
	44.9 (27.4–68.3)	48.6 (32.3–72.5)	
LPA, min/day	296.4 (85.6)	386.3 (78.6)	**<0.0001**
	291.6 (235.1–347.4)	382.3 (333.8–435.4)	
ST, min/day	470.3 (110.5)	421.9 (100.0)	**<0.0001**
	470.8 (398.8–549.1)	419.2 (358.5–487.9)	
Grip strength, kg	36.1 (5.5)	23.0 (3.8)	**<0.0001**
	36.3 (32.2–39.7)	23.0 (20.6–25.7)	
One-leg standing, s	85.5 (42.3)	81.7 (42.1)	0.59
	118.7 (46.8–120.0)	100.9 (40.0–120.0)	
Usual walking speed, m/s	1.45 (0.26)	1.48 (0.24)	**0.04**
	1.44 (1.27–1.61)	1.47 (1.32–1.62)	
Maximum walking speed, m/s	1.98 (0.36)	1.87 (0.27)	**<0.0001**
	1.95 (1.76–2.16)	1.85 (1.68–2.04)	
Chair-standing time, s	8.89 (2.59)	9.17 (2.85)	0.20
	8.57 (6.95–10.28)	8.87 (7.15–10.55)	

Bold font means *p* < 0.05. All values are mean (SD) and numbers of population (%). PA and PF values are mean (SD) and median (IQR), and the *p*-values were calculated by *t*-test. The *p*-values of frequency (numbers of individuals) were calculated by the Chi-square test.

**Table 2:** Associations between the quartiles of PA variables without adjustments and PF variables in the men.

	Q1	Q2	Q3	Q4	*p* for Trend
**MVPA**					
GS, kg	35.2 (5.8)	36.3 (5.8)	35.9 (5.7)	37.0 (4.8)	**0.04**
OS, s	72.3 (45.8)	92.2 (39.6)	87.2 (40.4)	90.5 (40.9)	**0.01**
UWS, m/s	1.35 (0.23)	1.42 (0.27)	1.47 (0.27)	1.55 (0.24)	**<** **0** **.0001**
MWS, m/s	1.85 (0.38)	1.99 (0.33)	2.03 (0.36)	2.04 (0.31)	**<0.0001**
CT, s	9.48 (2.67)	9.17 (2.31)	8.74 (2.60)	8.14 (2.62)	**<0.0001**
**LPA**					
GS, kg	35.5 (5.4)	36.0 (6.0)	37.3 (5.6)	35.6 (4.9)	0.50
OS, s	77.0 (44.7)	87.9 (41.5)	91.2 (39.0)	86.1 (43.3)	0.20
UWS, m/s	1.44 (0.28)	1.40 (0.22)	1.47 (0.27)	1.48 (0.27)	0.15
MWS, m/s	1.99 (0.42)	1.96 (0.33)	2.00 (0.35)	1.96 (0.31)	0.86
CT, s	8.84 (2.69)	8.80 (2.55)	8.75 (2.47)	9.15 (2.69)	0.43
**ST**					
GS, kg	36.2 (5.0)	36.3 (5.9)	36.1 (5.7)	35.9 (5.6)	0.61
OS, s	88.8 (40.9)	78.4 (45.2)	89.7 (41.2)	85.2 (41.5)	0.87
UWS, m/s	1.51 (0.27)	1.42 (0.26)	1.45 (0.27)	1.42 (0.25)	0.06
MWS, m/s	2.01 (0.32)	1.94 (0.31)	2.01 (0.38)	1.95 (0.40)	0.52
CT, s	8.87 (2.26)	9.18 (2.95)	8.68 (2.49)	8.83 (2.65)	0.48

Bold font means *p* < 0.05. All values are mean (SD). MVPA (min); Q1: MVPA < 27.40 (*n* = 97), Q2: 27.40 ≤ MVPA < 44.87 (*n* = 97), Q3: 44.87 ≤ MVPA < 68.31 (*n* = 97), Q4: 68.31 ≤ MVPA (*n* = 97). LPA (min); Q1: LPA < 235.13 (*n* = 97), Q2: 235.13 ≤ LPA < 291.63 (*n* = 97), Q3: 291.63 ≤ LPA < 347.41 (*n* = 97), Q4: 347.41 ≤ LPA (*n* = 97). ST (min); Q1: ST < 398.80 (*n* = 97), Q2: 398.80 ≤ ST < 470.77 (*n* = 97), Q3: 470.77 ≤ ST < 549.13 (*n* = 97), Q4: 549.13 ≤ ST (*n* = 97). CT: chair-standing time, GS: grip strength, MWS: maximum walking speed, OS: one-leg standing, UWS: usual walking speed.

**Table 3:** Associations between the quartiles of PA variables without adjustments and PF variables in the women.

	Q1	Q2	Q3	Q4	*p* for Trend
**MVPA**					
GS, kg	23.1 (4.1)	22.8 (3.9)	23.5 (3.7)	22.4 (3.6)	0.39
OS, s	78.1 (43.8)	83.4 (41.9)	82.5 (41.8)	82.6 (41.1)	0.27
UWS, m/s	1.39 (0.23)	1.45 (0.21)	1.54 (0.22)	1.54 (0.26)	**<0.0001**
MWS, m/s	1.81 (0.28)	1.82 (0.23)	1.92 (0.26)	1.92 (0.29)	**0.0002**
CT, s	9.62 (3.21)	9.32 (2.87)	9.01 (2.51)	8.75 (2.72)	**0.03**
**LPA**					
GS, kg	22.8 (4.0)	23.4 (3.6)	23.0 (4.0)	22.6 (3.8)	0.61
OS, s	78.4 (41.7)	82.1 (42.9)	82.1 (42.7)	84.2 (41.4)	0.48
UWS, m/s	1.48 (0.21)	1.47 (0.24)	1.48 (0.27)	1.49 (0.23)	0.66
MWS, m/s	1.85 (0.25)	1.89 (0.27)	1.85 (0.28)	1.88 (0.28)	0.70
CT, s	9.21 (2.68)	9.03 (2.58)	9.43 (3.00)	9.01 (3.12)	0.56
**ST**					
GS, kg	22.6 (3.9)	22.7 (3.8)	23.4 (3.2)	23.2 (4.3)	0.14
OS, s	84.1 (41.4)	86.0 (39.8)	81.7 (43.7)	75.0 (43.0)	0.13
UWS, m/s	1.49 (0.20)	1.50 (0.23)	1.47 (0.28)	1.47 (0.23)	0.46
MWS, m/s	1.87 (0.27)	1.87 (0.25)	1.86 (0.28)	1.86 (0.28)	0.78
CT, s	9.20 (2.62)	9.19 (3.13)	9.07 (2.84)	9.24 (2.81)	0.98

Bold font means *p* < 0.05. All values are mean (SD). MVPA (min); Q1: MVPA < 32.25 (*n* = 104), Q2: 32.25 ≤ MVPA < 48.63 (*n* = 108), Q3: 48.63 ≤ MVPA < 72.50 (*n* = 104), Q4: 72.50 ≤ MVPA (*n* = 106). LPA (min); Q1: LPA < 333.75 (*n* = 105), Q2: 333.75 ≤ LPA < 382.34 (*n* = 106), Q3: 382.34 ≤ LPA < 435.43 (*n* = 106), Q4: 435.43 ≤ LPA (*n* = 105). ST (min); Q1: ST < 358.50 (*n* = 105), Q2: 358.50 ≤ ST < 419.19 (*n* = 106), Q3: 419.19 ≤ ST < 487.88 (*n* = 106), Q4: 487.88 ≤ ST (*n* = 105). CT: chair-standing time, GS: grip strength, MWS: maximum walking speed, OS: one-leg standing, UWS: usual walking speed.

**Table 4:** Differences in physical function (PF) by quartile of MVPA after adjustments.

	Men (*n* = 388)					Women (*n* = 422)				
	Q1	Q2	Q3	Q4	*p* for Trend	Q1	Q2	Q3	Q4	*p* for Trend
**GS, kg**										
Model 1	35.3 (0.6)	36.2 (0.5)	35.9 (0.6)	36.9 (0.6)	0.26	23.4 (0.4)	22.8 (0.4)	23.4 (0.4)	22.3 (0.4)	0.13
Model 2	35.7 (0.6)	36.4 (0.5)	35.8 (0.5)	36.5 (0.6)	0.67	23.6 (0.4)	22.8 (0.4)	23.3 (0.4)	22.2 (0.4)	**0.045**
**OS, s**										
Model 1	78.9 (4.2)	89.9 (4.0)	87.6 (4.1)	85.7 (4.1)	0.29	81.8 (3.9)	85.0 (3.8)	81.0 (3.9)	78.9 (3.8)	0.80
Model 2	80.3 (4.2)	90.8 (4.0)	87.6 (4.0)	83.5 (4.1)	0.30	83.2 (3.9)	85.4 (3.7)	80.3 (3.8)	77.7 (3.8)	0.70
**UWS, m/s**										
Model 1	1.38 (0.03)	1.41 (0.03)	1.47 (0.03)	1.53 (0.03)	**<0.001**	1.41 (0.02)	1.46 (0.02)	1.53 (0.02)	1.53 (0.02)	**<0.001**
Model 2	1.40 (0.03)	1.42 (0.02)	1.47 (0.02)	1.50 (0.02)	**0.016**	1.42 (0.02)	1.46 (0.02)	1.53 (0.02)	1.52 (0.02)	**0.001**
**MWS, m/s**										
Model 1	1.90 (0.04)	1.98 (0.03)	2.02 (0.03)	2.01 (0.03)	0.09	1.82 (0.03)	1.83 (0.02)	1.91 (0.03)	1.90 (0.02)	**0.02**
Model 2	1.92 (0.03)	1.99 (0.03)	2.02 (0.03)	1.97 (0.03)	0.24	1.83 (0.02)	1.83 (0.02)	1.91 (0.02)	1.89 (0.02)	**0.03**
**CT, s**										
Model 1	9.19 (0.27)	9.20 (0.25)	8.89 (0.26)	8.26 (0.26)	**0.008**	9.41 (0.28)	9.25 (0.27)	9.14 (0.27)	8.89 (0.27)	0.63
Model 2	8.96 (0.26)	9.15 (0.24)	8.95 (0.24)	8.48 (0.25)	0.10	9.12 (0.26)	9.15 (0.25)	9.34 (0.26)	9.08 (0.25)	0.67

Bold font means *p* < 0.05. All PF values are multivariable-adjusted means (SE) and *p*-values for trend. The PF variables were used in Model 1 and Model 2, respectively. Model 1: Adjusted for age, BMI, self-rated health, smoking habit, alcohol intake, osteoporosis, hypertension, hyperlipidemia, diabetes, stroke, heart disease, years of education, and accelerometer-wearing time. Model 2: Adjusted for Model 1 and significant PF variables among the men and women, respectively. Because UWS and MWS were closely correlated with each other, they were not adjusted by each walking speed. Additionally, if other PC variables had significance for both UWS and MWS, they were adjusted only by UWS. CT: chair-standing time, GS: grip strength, MWS: maximum walking speed, OS: one-leg standing, UWS: usual walking speed.

## Data Availability

The statistical analyses were carried out using the computer resources offered under the category of General Projects by the Research Institute for Information Technology, Kyushu University, Japan.
